# The HIV epidemic and sexual and reproductive health policy integration: views of South African policymakers

**DOI:** 10.1186/s12889-015-1577-9

**Published:** 2015-03-04

**Authors:** Diane Cooper, Joanne E Mantell, Jennifer Moodley, Sumaya Mall

**Affiliations:** Women’s Health Research Unit, School of Public Health and Family Medicine, University of Cape Town, Anzio Road, Observatory, Cape Town, 7925 South Africa; School of Public Health, University of Western Cape, Private Bag X17, Bellville, 7535 South Africa; HIV Center for Clinical and Behavioral Studies, Division of Gender, Sexuality, and Health, New York State Psychiatric Institute and Columbia University, New York, NY USA; Cancer Research Initiative, Faculty of Health Sciences, University of Cape Town, Cape Town, South Africa; Department of Psychiatry and Mental Health, University of Cape Town, Cape Town, South Africa

**Keywords:** HIV/AIDS, Sexual and reproductive health, Integration, Policymakers, South Africa, Resourced-constrained settings

## Abstract

**Background:**

Integration of sexual and reproductive health (SRH) and HIV policies and services delivered by the same provider is prioritised worldwide, especially in sub-Saharan Africa where HIV prevalence is highest. South Africa has the largest antiretroviral treatment (ART) programme in the world, with an estimated 2.7 million people on ART, elevating South Africa’s prominence as a global leader in HIV treatment. In 2011, the Southern African HIV Clinicians Society published safer conception guidelines for people living with HIV (PLWH) and in 2013, the South African government published contraceptive guidelines highlighting the importance of SRH and fertility planning services for people living with HIV. Addressing unintended pregnancies, safer conception and maternal health issues is crucial for improving PLWH’s SRH and combatting the global HIV epidemic.

This paper explores South African policymakers’ perspectives on public sector SRH-HIV policy integration, with a special focus on the need for national and regional policies on safer conception for PLWH and contraceptive guidelines implementation.

**Methods:**

It draws on 42 in-depth interviews with national, provincial and civil society policymakers conducted between 2008–2009 and 2011–2012, as the number of people on ART escalated. Interviews focused on three key domains: opinions on PLWH’s childbearing; the status of SRH-HIV integration policies and services; and thoughts and suggestions on SRH-HIV integration within the restructuring of South African primary care services. Data were coded and analysed according to themes.

**Results:**

Participants supported SRH-HIV integrated policy and services. However, integration challenges identified included a lack of policy and guidelines, inadequately trained providers, vertical programming, provider work overload, and a weak health system. Participants acknowledged that SRH-HIV integration policies, particularly for safer conception, contraception and cervical cancer, had been neglected.

Policymakers supported public sector adoption of safer conception policy and services. Participants interviewed after expanded ART were more positive about safer conception policies for PLWH than participants interviewed earlier.

**Conclusion:**

The past decade’s HIV policy changes have increased opportunities for SRH–HIV integration. The findings provide important insights for international, regional and national SRH-HIV policy and service integration initiatives.

## Background

International conferences such the International Conference on Population and Development in 1994 and international bodies such as the World Health Organisation and the United Nations through its programme on HIV and AIDS have promoted an integrated and comprehensive approach to HIV health service provision at primary care level [1-8]. Service integration consists of bringing together different activities that share common goals [4]. This can occur at a number of different levels – policy planning, finance, governance, service provision and monitoring and evaluation [9,10]. Policies to integrate HIV activities have occurred across a range of policy areas and in a variety of settings-within contraceptive, sexually transmitted infections (STI) and maternal health services as well as outside of these settings, for example, between HIV and tuberculosis (TB) [2]. In South Africa, policymakers have focused on policies, guidelines and services to integrate HIV and TB, and HIV and STI, since 2003 [9].

Public sector health policymakers and civil society leaders are likely to have strong opinions about developing, integrating and prioritising sexual and reproductive health (SRH)-HIV policies, including safer conception and contraception into HIV services. These stakeholders play a central role in influencing policies, guidelines, provider training and provision for SRH and HIV services [11,12]. There are strong arguments for policy, programmes and service integration for SRH and HIV as both focus on human sexuality and dual risks of unintended pregnancy and STI. Closer links between HIV and SRH can help decrease the likelihood of missed opportunities for providing SRH care, offering a broader range of services [7].

South Africa has approximately 6.4 million (12% of the overall population) people living with HIV (PLWH) [10] and a sizable proportion of women of reproductive age living with HIV are on antiretroviral treatment (ART) [13,14]. The country provides an excellent context to address challenges to SRH and HIV policies and services integration, reflecting “a microcosm of impediments to improving population health globally” [15], particularly with respect to HIV. A number of key policy changes have occurred in SRH and HIV. These include changes in ART and Prevention of Mother-to-Child-Transmission of HIV (PMTCT) (vertical transmission) policies and in criteria for ARV initiation [16]; introduction of provider-initiated HIV counselling and testing; and from 2007, massive expansion of public sector ART. In 2011, the South African government re-committed itself to SRH rights [17], articulating the need for specific policies on reproductive choices for PLWH. The 2011 National Health Insurance Green Paper (proposed law), being piloted in 10 districts across all provinces over the next 14 years, is aimed at redressing broader health system weaknesses and inequities including that of SRH and HIV care [18].

While the government has encouraged primary care policy and service integration since 1994, HIV policy and services have taken a vertical approach. Policies and services for HIV and TB, STIs and maternal health have been substantially integrated over the past 8 years, but with the recent exception of contraception and HIV, SRH-HIV policy and service integration has not occurred. In June 2014, the National Department of Health launched the 2012 revised National Contraception and Fertility Planning Policy and Service Delivery Guidelines. These outline provisions for contraception for PLWH, considering factors such as age, fertility intentions, disease stage, transmission risk, progression, and acquisition and interactions between ART medications and hormonal contraception [19]. Other salient features include promoting an expanded method mix through long-acting contraceptive methods, such as intra-uterine devices (IUDs) and sub-dermal hormonal implants, and increasing access to female condoms and emergency contraception [19]. More recently, the National Department of Health has cautioned the use of implants in women living with HIV (WLWH) for contraception due to possible interactions with a key antiretroviral drug, Efavirenz [20].

Four years ago, the Southern African HIV Clinicians Society published safer conception guidelines for PLWH in both resource-intense and resource-limited settings [21]. While the concept of promoting fertility planning for PLWH was incorporated into the 2012 national fertility planning guidelines for contraception, to date there is no specific South African government or regional policy or guidelines on safer conception choices and services for PLWH. The 2002 National Cervical Cancer Screening Policy did not make provision for screening WLWH [16], but is being revised to take this into account. The National Department of Health has issued guidelines for cervical cancer screening in HIV-positive women from age 20, and thereafter, every 3 years [16]. Some services have been screening WLWH more frequently, but uniform practices need to be implemented. Failure to address the need for integrated SRH-HIV policies and services has negative public health implications for unintended pregnancies among WLWH, secondary HIV transmission from an HIV-infected to an uninfected partner when trying to conceive, and for early detection and treatment of cervical cell abnormalities and cervical cancer (an AIDS-defining illness) in WLWH [2,5,13,21,22]. The absence of policies that tailor these specific SRH services to PLWHs’ needs leaves a hiatus, especially in high HIV prevalence, resource-limited settings [23,24].

Recognising the critical need for policies and guidelines for greater integration of primary care SRH and HIV services, we conducted a study of South African health policymakers and key civil society informants’ views on the feasibility and possible pathways for SRH integration into HIV care services, with particular focus on policy for contraception and safer conception for PLWH. The majority of the data was collected as part of formative research to inform the design of a structural intervention to integrate SRH and HIV care into public sector services in Cape Town. A few interviews were conducted after the intervention was implemented. The aim of the qualitative research was to explore the context in which the intervention was to be implemented and inform the design of the intervention. Exploring policymakers’ views is useful to inform the development and implementation of policies on integration of HIV, contraception and safer conception for PLWH, and is particularly timely given the expansion of ART in southern Africa over the past 5 years.

## Methods

We used a qualitative research design for in-depth exploration of how stakeholders viewed health system integration and their experiences of previous integration efforts. A core objective of qualitative research is to uncover how people understand their ‘life worlds’ and make meaning of everyday phenomena. This approach was chosen as appropriate in fulfilling the study objectives to enquire, analyse and understand participants’ subjective meanings about policy and service integration within their social and health system contexts [25] and refine the SRH-HIV intervention study component prior to its implementation [26,27].

### Population and sample

In order to obtain diversity of opinions, we recruited a purposive sample of 42 South African policymakers in public and civil society sectors in HIV and SRH, at both a national and provincial level. The Western Cape and Gauteng Provinces were selected because their HIV service models have frequently been incorporated into national HIV policy. Thirty-seven interviews were conducted between January 2008 and June 2009, and an additional five interviews were conducted towards the end of the study in late 2011/early 2012 with national participants. Once saturation of data had occurred, and the interviewees were no longer providing new information, no further interviews were conducted.

We explained study objectives and procedures and initiated an informed consent process with prospective participants. No one refused to be interviewed. Participants chose the location and time/day of the interview.

Interviews were conducted in English by three senior researchers experienced in qualitative interviewing using a semi-structured interview guide. Interviews focused on three key domains: opinions on PLWH’s childbearing; current status of sexual and reproductive and HIV integration policies and service integration; and thoughts and suggestions about SRH-HIV integration within the context of the restructuring of South African primary care services. In addition, we collected demographic data on participants.

Interviews lasted between one-and-a half and two hours. All interviews were audio-recorded and transcribed verbatim. Interviewers kept notes of thoughts during the research process in order to forefront their own opinions and biases.

### Analysis

For analysis, initial coding categories for segments of transcribed text were drawn from the interview guides. In addition, we were attentive to our field notes to inform the codes and categories emerging from the data. Codes relating to the three key domains were developed. The first author synthesised the coding list, which was reviewed by the other three authors. Two members of the research team independently coded transcripts and codes were then discussed to resolve any coding discrepancies and ensure common interpretation. Codes were entered into NVivo 9 to facilitate data sorting and management. An inductive, thematic analysis approach was used to generate exploratory, descriptive themes emerging from the interviews. Further sub-themes were identified to provide a rich and comprehensive account of the data [28]. For reliability, a fourth researcher independently checked data and themes. Participants were offered their individual interview transcript to check. None chose to comment on transcripts.

Illustrative quotations relevant to each theme were extracted. The quotations selected from the raw data were typical of participants interviewed and therefore were dominant themes, unless otherwise specified. Alternative views were sought in coding the data and extracting themes; where these were evident, they are presented in the data as divergent or minority views. Narratives and themes were compared to assess similarities and differences between participants. Possible alternative explanations for findings were examined.

### Ethics

The study protocol, interviews, and consent forms were reviewed and approved by the Institutional Review Board at the New York State Psychiatric Institute and Columbia University Department of Psychiatry, and the Health Sciences Faculty Human Research Ethics Committee at the University of Cape Town. Permission to conduct the study was obtained from the relevant health authorities. All participants completed written informed consent forms prior to being interviewed. Anonymity in reporting and confidentiality of data were discussed with participants and assured. Where possible, participant professional categories were noted for each quotation. However, more general labels were given to quotations where there was a single or few individuals in a category to safeguard participant anonymity.

## Results

This section describes the study participants’ background; views on PLWH’s childbearing; opinions on policies and levels of integration of SRH within HIV/AIDS care and treatment; attitudes towards SRH-HIV integration; and lessons learned from other experiences, with a special emphasis on safer conception policies for PLWH.

### Background of participants

The 42 participants comprised 19 national, provincial and local government policymakers and managers, 15 civil society organisation (CSO) and Non-Governmental Organisation (NGO) leaders, 4 academic researchers, and 4 dually-appointed government health department and academic HIV specialist physicians. Years of work in SRH or HIV ranged from 1 to 20. Participants were between 30 and 50 years of age. Twenty-four were women and 18 were men. Some participants had moved between posts in different sectors and health policy areas, giving them a range of perspectives on health policy integration.

Participants’ perspectives on SRH-HIV care integration centred on four overall themes that emerged from questions in the interview guide: (1) inadequacy of current policy, guidelines and services for SRH-HIV integration; (2) integration advantages and disadvantages and support in principle for improved SRH-HIV service integration but caution and challenges; (3) lessons to be learned for SRH-HIV integration policies from other integration initiatives; and (4) specific views on childbearing in PLWH and integration of safer conception for PLWH into policy.

### Inadequacy of current policies on SRH-HIV integration

Participants initially perceived integration to mean providing clients with a comprehensive care package. In addition, policymakers’ views on SRH-HIV integration were interpreted as narrowly focusing on safer sex issues. When probed about other aspects of SRH, participants noted an absence of HIV-SRH integration policies:

*Sexual and reproductive health policies* [relating to HIV] *are bad…and I think that… guidelines have lagged behind the science.* [Doctor, Academic Hospital, National Key Informant, 2008/9].

*...there’re some practical obstacles like, you know, you need the* [policy] *document that talks to the vision* [on integration] *and the way it’s going to be done and so on…it doesn’t exist.* [Provincial Health Department of the Western Cape (WCDoH) Policymaker, 2008/9]*.*

A policymaker from the City of Cape Town Health Department reiterated others’ views in observing lack of clarity and policy on cervical screening for WLWH, for example:

“...*general cervical screening policy which is.. a pap smear every 10 years from 30* [years] *onwards, does not really apply to HIV+ women, ...as far as I know, I could be wrong, there’s not an official policy to say what would be the best time to repeat the pap smear for a* [HIV+] *woman, but I know a lot of our services actually just do it annually, so the HIV+ women do have an opportunity to access Pap smears more frequently than someone that’s not HIV”*. [CCTDoH District Health Manager/Policymaker, 2008/9].

### SRH-HIV integration’s advantages and disadvantages

There was substantial support for policy on broader integration of SRH-HIV health services as defined earlier, including contraception, pregnancy and maternal health, cervical screening and other STIs. Independent of existing policy, policymakers were aware of ad hoc efforts at SRH-HIV integration at primary healthcare level, but not at specialised, tertiary level HIV services.

There were divergent views on tailoring SRH services to the needs of women living with HIV. Some participants viewed WLWH’s SRH needs as unique, whereas others perceived them to be similar to those of uninfected women. Participants in the latter group cautioned against policy separating services based solely on HIV+ status. However, all saw key advantages to integrated care in that there would be fewer missed opportunities to address unintended pregnancies, potentially improved pregnancy and maternal health outcomes, and attention to WLWH’s cervical health issues.

Participants emphasised the specificity of service delivery context:

***…****I think there’s not one size fits all. So yes, in some places there might be advantages … but like in the bigger, busier sites, it is more efficient to have one somebody, who might be doing Pap smears all day, for instance, as opposed to one somebody who’s doing anything that walks in the door that might be general HIV and STI and at the same time if they need a Pap smear, and she does the Pap smear…”*. [WCDoH Provincial Policymaker, 2008/9].

### Lessons from other integration initiatives and suggestions for SRH-HIV integration

Shortcomings in prior policy, guidelines and services integration implementation strongly underscored the importance of healthcare providers understanding and prioritising their leadership and involvement in integration efforts:

*So if it’s not provider initiated, you’re going to land up with people only having little segments of information. Now if you are trying to integrate reproductive health, HIV, TB, STI, by virtue of integrating it,* [you] *have given that person a broader knowledge base, which …empowers them to have a broader sense of choice.* [WCDoH Policymaker, 2008/9].

Participants mentioned experiences with maternal health services and TB care with HIV care integration as key points of reference for further integration initiatives. There was a general consensus that while PMTCT was highly successful in reducing infant HIV infection, overburdening providers by integrating more SRH services into HIV care could increase their workload:

*We found that the demands of the PMTCT programme were very high on service deliverers, and therefore to bring family planning into that as well was just adding to their workload. It was difficult to coordinate times and visits, but it was nevertheless part of their agenda, and they did do some of it….*[Researcher in Child Health, Academic Institution, Western Cape, 2008/9].

Several policymakers noted that until very recently PMTCT had a strong focus on preventing infant HIV transmission but insufficiently addressed pregnant WLWH’s needs. Most participants mentioned high levels of maternal mortality among women who had AIDS.

TB was seen as an exemplar policy model for integration. Policy promoting TB-HIV integration promoted service provision in which providers proactively ask HIV-positive clients about possible TB symptoms. Policymakers felt that policy promoting integration of contraception and safer conception in particular, could have a similar spin-off in providers incorporating routine practices to encourage fertility planning among women and men living with HIV and couples.

### Views on biological children and safer conception policies for PLWH

No participants expressed views that PLWH should not have biological children. They noted the importance of viewing childbearing in PLWH in a non-judgmental manner and reported that some providers were judgmental about this. Nevertheless, participants expressed greater comfort with PLWH having children when they were on ART and achieved viral suppression. They thought that it would be easier to incorporate discussion of childbearing choices at this time:

*So the one scenario is the HIV+ woman who’s presenting and you know she’s had a previous* [pregnancy], *she’s on ARVs* [antiretrovirals]*… and you know her viral load is suppressed and she had a previous child who passed away, I’d be very supportive of* [a policy on] *something like… having… the pregnancy and the baby and all those things.* [WCDoH Policymaker, 2008/9].

In 2008/09, participants did not mention safer conception for PLWH unprompted. When this issue was probed, most participants were uncertain about possible options. Only one national public sector policymaker mentioned the need for such policy in the context of expanding ARV treatment and no specific policy recommendations were made:

*Ja, I would say we should be considering safer conception policy for HIV+ people. It makes sense now that ARVs are being rolled out. I am in favour if people are on ARVs, but we are worried if they are not on ARVs. I know some of the methods but our knowledge is not enough at our level, let alone at service level. There is really no policy or guidelines. So many policymakers and providers do not have good knowledge of what safer conception methods are possible for those living with HIV. We need to spell this out in policy and have providers feeling this is a good idea…I think most would, but there are still some who are reluctant, particularly if the woman is negative* [and the male is HIV-positive]….” [National Policymaker, Maternal Health and Reproductive Health, NDoH, 2009].

In interviews in 2011/12, while not all participants were knowledgeable about the full content of the 2011 Southern African HIV Clinicians’ Society safer conception guidelines, all were aware that new methods for safer conception for PLWH existed and were feasible in resource-limited contexts. They were keen on policy and guidelines being developed and implemented and mentioned some specific safer conception methods:

*I think there is need for policy and guidelines. What we would be willing to do and what not. For example, there is talk of giving PrEP to HIV-negative women whose male partners are positive as a targeted population. We can’t afford PrEP for everyone. But if this can help couples plan a pregnancy, it could really help* [to give PrEP to HIV-negative women of HIV-positive partners wishing to conceive] *for women not to become infected. For example, we have a real problem with women only sero-converting during pregnancy and it not being picked up. This causes problems with maternal health and with infant transmission.* [National Policymaker, HIV, NDoH, 2012].

A participant echoed the views of others in suggesting that new biomedical prevention and treatment methods could help HIV-positive people wishing to have biological children:

*...I would be in favour of policy that lists all the methods, including this one for couples where the man is on ARVs and the women not infected at first* [having ARVs available for her]*. We could get to the women through the men….* [National Policymaker, HIV, NDoH, 2012].

However, implementation of new initiatives in this regard seemed to be limited to research organisations:

*We are now giving counselling and demonstrations on safer conception methods, especially manual self-insemination with an uninfected male partner’s sperm for women living with HIV. They are so keen that we are trying to introduce a service.* [HIV Clinician, Academic Research Institution, Western Cape, 2011].

*We are running a clinic for PLWH who want to become pregnant in Gauteng. I have assembled all the counselling materials and guidelines. This is for those on ARVs, but I see anyone with HIV. I can’t keep up with the huge demand.* [HIV and SRH Clinician, Academic Research Institution, Gauteng, 2012].

Government policy and services on counselling and safer conception methods for PLWH seeking conception have not been formulated. One national policymaker echoed others’ views in stating:

*No policy currently talks about women and men conceiving more safely when HIV+. We have veered away from this and this needs to change with ARV roll-out…We were reluctant in the pre-ARV days.* [National Policymaker, NDoH, 2012].

Another reiterated this view in saying:

*Five years ago we had no idea what to do to make becoming pregnant safer for clients living with HIV. There are a lot of new ideas with ARVs, even for those not on ARVs at the moment and we will be moving in the future to PMTCT B +. Now we do know what we can do. Things have changed so much, but we still have no national policy or guidelines…* [National Policymaker, SRH and Women’s and Maternal Health, NDoH, 2012].

## Discussion

Much has been written in the scientific literature about health service integration, and most view integration positively [29,30]. Studies have examined integration of HIV messaging into contraceptive services [9,31] and more recently contraception into HIV care [32]. Some studies in southern and east Africa have shifted from examining reproductive intentions of PLWH [33-38] to exploring client and provider views on SRH-HIV integration [39,40]. Few have examined policymakers’ views, which are so critical to any changes in policy and service delivery [41]. Hence, this study contributes to production of knowledge by offering new insights into policymakers’ views on integration.

Participants’ views on biological children for PLHW may have been coloured in some cases by political correctness. However, their views that in practice PLWH were having children intentionally and unintentionally coincide with other limited policymaker research [41]. Participants were more comfortable with policies promoting safer conception counselling and services for ART clients, particularly if they had no children. Policymakers interviewed in 2011/2012 showed positive attitudes towards newer effective and acceptable safer conception methods for PLWH [42,43]. Participants’ belief that demand for such services escalates once clients are aware of their existence resonates with research on providers’ [44,45] and clients’ views about safer conception [46]. In addition, they suggested that should PLWH wish to avoid a pregnancy, HIV carers should counsel and provide them with a contraceptive method of their choice. Their views that providers tend to hold judgmental attitudes towards PLWH having children, concurs with other studies [34,39]. While negative attitudes to PLWH childbearing may be shifting in light of accelerated ART, changing judgmental attitudes towards reproduction in PLWH, particularly at provider level, is important [34,39-41]. As indicated in our findings, policymakers are keen to develop SRH-HIV integration policies and plan developing services with managers and providers.

As other studies on SRH-HIV in South Africa [41] and HIV cervical screening integration in Uganda found [47], experience with past integration initiatives are important in considering new initiatives. Participants described facilitators to integration as: healthcare providers understanding of policies; prioritisation of provider leadership and involvement in integration efforts; and avoidance of provider work overload when more tasks are added to an overburdened public healthcare system. A few stressed the need for trained healthcare providers providing SRH counselling and services within HIV care, particularly on safer conception. Policymakers’ concerns about the potential for provider work overload in integrated services are underscored by core weaknesses and inefficiencies in the health system, including insufficient human resources. These issues are not unique to HIV care. The South African National Health Insurance proposed programme presents golden opportunities to strengthen the overall health system. A strengthened health system could promote effective SRH-HIV care integration efforts for PLWH. The emphasis on training and quality of care by the National Health Insurance programme [18] could be used as a motivation for values clarification training to counter healthcare providers’ judgmental attitudes towards PLWH’s childbearing. In a later phase of our study (from 2010 to 2012), we developed and implemented an intervention to integrate SRH counselling and services. We trained and provided on-going mentoring and support to NGO peer-counsellors and nurses integrating SRH and HIV within the public health sector and facilitated contraceptive provision within HIV care and referral for safer conception. Analysis of the intervention’s impact on reproductive health outcomes is underway and could provide a useful basis on which to consider expanded integrated SRH-HIV healthcare delivery.

Participants indicated that policy development and SRH-HIV integration were currently limited. This supports other studies showing that with the exception of safer sex messages and condom provision, SRH-HIV integration interventions are only being piloted in research projects [41,44,48,49]. Despite new contraceptive policy and guidelines in 2012 promoting contraceptive-HIV integration, implementation of this policy and guidelines needs evaluation. The publication of the Southern African HIV Clinicians Society’s safer conception guidelines 4 years ago was ground breaking in advising on PLWH’s safer conception choices in resource-limited settings [21]. However, there has been virtually no progress in national and regional governments’ translation of these guidelines into clinical practice in the public sector. South Africa has introduced specific integration policies on PMTCT [50], PEP [51] and ART [16] and updated ART guidelines [52] for PLWH. Given this, it is timely for government to effectively implement HIV-related contraceptive and develop feasible safer conception policies, guidelines and services. This has policymaker support and could address HIV care providers’ frustrations at being ill equipped to counsel PLWH on safer conception [40]. Consideration should be given to including PrEP for discordant couples planning to conceive, where the woman is HIV-negative [53]. Participants and recent studies indicate research initiatives implementing the Southern African HIV Clinicians Society publication of guidelines in providing counselling and safer conception services in the Western Cape and Gauteng [41,44]. These research endeavours should be evaluated and built on for feasibility in the public sector and regional expansion.

While slow public sector adoption of guidelines and health system programmes contribute to limited progress on SRH-HIV integration, international and national funding for vertical HIV care over the past 10 years have compromised support for integrated, comprehensive SRH-HIV policies and services and tended to cause neglect of broader SRH issues.

Developing and implementing SRH-HIV specific policies, guidelines and services on their own will not remove all barriers to SRH rights and choices for men and women living with HIV, as socio-economic, interpersonal and individual factors also influence decision-making and service use [46]. However, they are an important first step to realising SRH rights and choices for PLWH and rendering comprehensive healthcare.

Researcher attitude bias is possible in every study. However, to reduce bias, qualitative research encourages self-reflexivity and acknowledges researchers’ influence on the research process. In conducting the research and analysis, researchers were aware and reflected on their own research roles and interpretations of the data. A further study limitation is that qualitative studies seek to obtain a range and depth of perceptions and attitudes and do not allow for generalisability. Qualitative research sets out to examine the ‘how’ rather than the ‘how much’ [25]. Nonetheless, a large number of policymakers were purposively selected and interviewed in this study and rich insights produced on integration of SRH and HIV and safer conception in particular, relevant for policies in South Africa and other countries with high HIV prevalence in sub-Saharan Africa.

## Conclusion

South Africa is recognised as having introduced some exemplary SRH-related laws and policies on rights for abortion and contraception over the past 20 years. Since 2009, it has made substantial progress for a high HIV-prevalence country, in preventing HIV vertical transmission and providing ART. Changes in HIV policy over the past 8 to 10 years provide opportunities for HIV-SRH and rights policy integration, particularly with regard to contraception, safer conception and cervical screening. These are important for international, regional and national health policy and SRH-HIV service integration initiatives [17,19,21,48,49]. Changing practice is difficult for policymakers, providers and clients. In adopting new policies, programmes and services, we need cooperation among policymakers, health providers, clients and their partners, and the broader community. A comprehensive and integrated approach to HIV and SRH that includes preventing unintended pregnancies and promoting safer conception, attending to women who seroconvert during pregnancy, identifying women with AIDS and other illnesses during pregnancy and childbirth [54], and implementing tailored cervical screening for WLWH, is crucial for respecting SRH rights and reproductive choices of PLWH and combatting the global HIV epidemic.

## Abbreviations

ARV(s): Antiretroviral(s)
ART: Antiretroviral Treatment
CCTDoH: City of Cape Town Department of Health
CSO: Civil Society Organisation
HIV: Human Immunodeficiency Virus
NDoH: National Department of Health
NGO: Non-Governmental Organisation
NHI: National Health Insurance
PEP: Post-exposure Prophylaxis for HIV
PLWH: People Living with HIV
PMTCT: Prevention-of-Mother-to-Child Transmission (vertical transmission) of HIV
PrEP: Pre-exposure Prophylaxis
SRH: Sexual and Reproductive Health
STI(s): Sexually Transmitted Infection(s)
TB: Tuberculosis
WCDoH: Western Cape Department of Health
WLWH(A): Women Living with HIV(/AIDS)
